# Gene Silencing of Angiopoietin-like 3 (ANGPTL3) Induced De Novo Lipogenesis and Lipid Accumulation in Huh7 Cell Line

**DOI:** 10.3390/ijms25073708

**Published:** 2024-03-26

**Authors:** Ilaria Rossi, Giorgia Marodin, Maria Giovanna Lupo, Maria Pia Adorni, Bianca Papotti, Stefano Dall’Acqua, Nicola Ferri

**Affiliations:** 1 Department of Pharmaceutical and Pharmacological Sciences, University of Padova, 35131 Padova, Italy; ilaria.rossi.11@phd.unipd.it (I.R.); giorgia.marodin@phd.unipd.it (G.M.); stefano.dallacqua@unipd.it (S.D.); 2 Department of Medicine, University of Padova, 35128 Padova, Italy; mariagiovanna.lupo@unipd.it; 3 Department of Medicine and Surgery, University of Parma, Via Volturno 39/F, 43125 Parma, Italy; mariapia.adorni@unipr.it; 4 Department of Food and Drug, University of Parma, Viale delle Scienze 27/A, 43124 Parma, Italy; bianca.papotti@unipr.it; 5 Veneto Institute of Molecular Medicine (VIMM), Via Orus, 2, 35129 Padova, Italy

**Keywords:** ANGPTL3, vupanorsen, lipids, PCSK9, de novo lipogenesis

## Abstract

Angiopoietin-like 3 (ANGPTL3) is a hepatokine acting as a negative regulator of lipoprotein lipase (LPL). Vupanorsen, an *ANGPTL3* directed antisense oligonucleotide, showed an unexpected increase in liver fat content in humans. Here, we investigated the molecular mechanism linking *ANGPTL3* silencing to hepatocyte fat accumulation. Human hepatocarcinoma Huh7 cells were treated with small interfering RNA (siRNA) directed to *ANGPTL3*, human recombinant ANGPTL3 (recANGPTL3), or their combination. Using Western blot, Oil Red-O, biochemical assays, and ELISA, we analyzed the expression of genes and proteins involved in lipid metabolism. Oil Red-O staining demonstrated that lipid content increased after 48 h of *ANGPTL3* silencing (5.89 ± 0.33 fold), incubation with recANGPTL3 (4.08 ± 0.35 fold), or their combination (8.56 ± 0.18 fold), compared to untreated cells. This effect was also confirmed in Huh7-LX2 spheroids. A total of 48 h of *ANGPTL3* silencing induced the expression of genes involved in the de novo lipogenesis, such as fatty acid synthase, stearoyl-CoA desaturase, ATP citrate lyase, and Acetyl-Coenzyme A Carboxylase 1 together with the proprotein convertase subtilisin/kexin 9 (PCSK9). Time-course experiments revealed that 6 h post transfection with *ANGPTL3-*siRNA, the cholesterol esterification by Acyl-coenzyme A cholesterol acyltransferase (ACAT) was reduced, as well as total cholesterol content, while an opposite effect was observed at 48 h. Under the same experimental conditions, no differences in secreted apoB and PCSK9 were observed. Since PCSK9 was altered by the treatment, we tested a possible co-regulation between the two genes. The effect of *ANGPTL3*-siRNA on the expression of genes involved in the de novo lipogenesis was not counteracted by gene silencing of *PCSK9*. In conclusion, our in vitro study suggests that *ANGPTL3* silencing determines lipid accumulation in Huh7 cells by inducing the de novo lipogenesis independently from PCSK9.

## 1. Introduction

Angiopoietin-like 3 (ANGPTL3) was first identified and cloned in 1999 [1], and its role in lipid metabolism was described in a subgroup of inbred strain KK obese mice (named KK/San) [2]. Liver-specific overexpression of ANGPTL3 or intravenous injection of the purified protein in KK/San and C57BL/6 mice determined an increase in circulating plasma lipid levels [2]. The link between ANGPTL3 and lipid metabolism is related to an impairment of very low-density lipoprotein triglycerides (VLDL-TG) clearance, due to the inhibition of lipoprotein lipase (LPL) activity [3] and by a direct activation of lipolysis in adipocytes [4], a process resulting in free fatty acid (FFA) and glycerol release into the circulation [4]. Beyond LPL, ANGPTL3 also inhibits the phospholipase activity of endothelial lipase (EL) [5], an expressed enzyme anchored on the luminal surface of endothelium that preferentially regulates plasma HDL cholesterol levels [5]. EL is more active as a phospholipase enzyme compared to hepatic lipase (HL) and LPL, which preferentially catalyzed the hydrolysis of TG [6]. Loss-of-function (LOF) mutations in the *ANGPTL3* gene determine familial combined hypolipidemia (FHBL2), a disorder characterized by very low levels of apolipoprotein B (apoB), apolipoprotein A1 (apoA-1) and their associated lipoproteins VLDL, low-density lipoprotein (LDL), and high-density lipoprotein (HDL), compared to non-carriers [7]. Heterozygous LOF variants in *ANGPTL3* were also associated with decreased odds of atherosclerotic cardiovascular disease [8]. Given the relationship between LOF mutations in *ANGPTL3* gene and the lower risk of developing ASCVD, a pharmacologic intervention aiming at reducing ANGPTL3 levels is promising. This evidence led to the development of pharmacological agents acting as ANGPTL3 inhibitors, namely the monoclonal antibody (mAb) evinacumab [9,10], and the N-acetyl galactosamine (GalNAc) modified oligonucleotide antisense (ASO) vupanorsen [11,12]. Evinacumab is currently approved for the treatment of patients with homozygous familial hypercholesterolemia, regardless of the degree of their LDL-receptor function [13]. Notably, the mechanism of action does not engage the LDL receptor; in fact, ANGPTL3 inactivation may lower LDL levels by reducing the liver’s secretion of VLDL particles, leading to a decreased processing of VLDL remnants into LDL [14]. The inactivation of ANGPTL3, either by genetic deletion or with a mAb, reduced the secretion of TG, but not of apoB-100 or apoB-48 [14,15]. Instead, the reduction in LDL and apoB, in response to ANGPTL3 inhibition, can be explained by the increased clearance of apoB-containing lipoproteins as they progress through the lipolytic cascade, thereby decreasing the fraction of VLDL that is converted to LDL and reducing LDL production [14]. This is consistent with the observation that the inactivation of *ANGPTL3* in mice, by gene targeting or by anti-ANGPTL3 antibodies, reduced plasma cholesterol levels in mice lacking functional ApoE [16] or LDL receptors [17]. Vupanorsen, the Gal-NAc-ASO, confirmed that the inhibition of *ANGPTL3* production leads to a significant reduction in non-HDL-cholesterol and TG, with a more modest effect on LDL-cholesterol and apoB, as seen from the phase 2b clinical trial TRANSLATE-TIMI 70 [18]. However, the administration of vupanorsen has resulted in an increase in liver enzymes and a dose-dependent elevation of fat in the liver. This increase is proportional to the effective silencing of *ANGPTL3* [18]. This effect has not been observed with the monoclonal antibody Evinacumab, which, on the contrary, has been shown to be well-tolerated by patients. Real-world studies have demonstrated that Evinacumab is a safe and effective treatment for these patients [19,20]. Genetically determined complete or partial absence of ANGPTL3 in human subjects does not correlate either with changes in liver fat content, hepatic steatosis risk, or variations in extrahepatic fat distribution [12,21]. Finally, no such effects were observed in *ANGPTL3* null mice [22].

Considering this apparent discrepancy, we decided to investigate the basic molecular mechanism underlying the lipid accumulation in hepatocytes by using an in vitro cultured cell line.

## 2. Results

**Figure 1:**
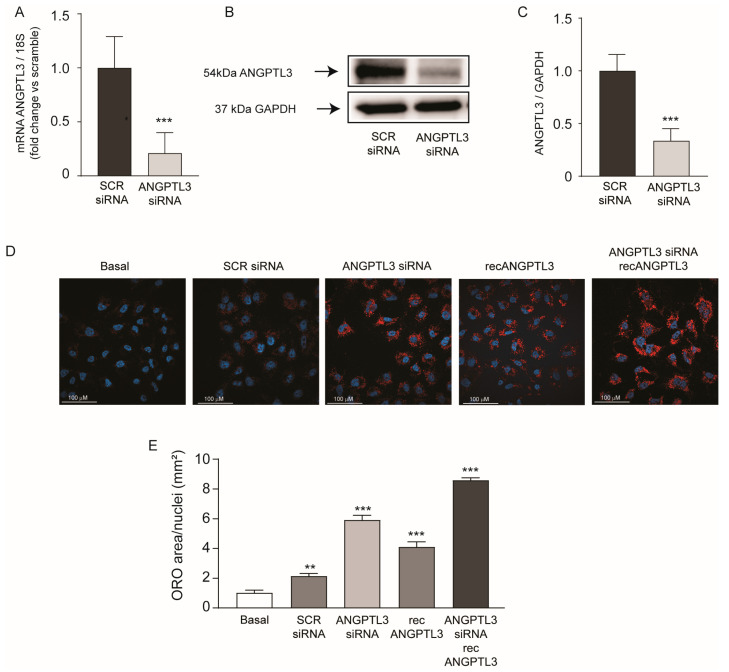
*ANGPTL3*-siRNA induced lipid accumulation in human hepatoma cell line Huh7. (**A**) RT-qPCR was performed on total RNA. Data expressed as ΔΔCt referred to cells transfected with siRNA-scramble. (**B**,**C**) ANGPTL3 expression was determined by Western blot analysis and GAPDH was used as the loading control. (**C**) Bar graphs of quantification of Western blot analysis. *p* values were calculated using Student’s *t*-test *** *p* < 0.001 vs. siRNA-scramble. (**D**) Neutral lipid content was visualized by Oil Red-O staining. Representative images are shown in panel (**D**), blue is DAPI (nuclei), red is Oil Red-O (neutral lipids). (**E**) Quantification of Oil Red-O area relative to nuclei was performed with ImageJ v.1.54d. *p* values were calculated using Student’s *t*-test. ** *p* < 0.01, *** *p* < 0.001 vs. basal. All data are presented as mean ± SD of three independent experiments.

In the first series of experiments, we explored the effect of *ANGPTL3* gene silencing on lipid accumulation in human hepatoma cell line Huh7. Commercially available small interfering RNA (*ANGPTL3*-siRNA) determined a significant reduction in mRNA (−79%) and protein levels (−67%) of ANGPTL3 compared to scramble-siRNA (Figure 1A–C). Lipid accumulation was then evaluated by Oil Red-O staining detecting neutral lipids, such as TG and cholesterol esters. Huh7 cells incubated with cultured media containing 10% FCS showed a minimal content of lipids under basal condition or after transfection with scramble-siRNA (Figure 1D). Differently, *ANGPTL3*-siRNA determined a significant increase in intracellular lipids (5.9 ± 0.3 fold). To resemble the physiological condition, we incubated the cells with 100 ng/mL of human recombinant ANGPTL3 in the absence or presence of *ANGPTL3*-siRNA [23]. Interestingly, ANGPTL3 also significantly induced intracellular lipid accumulation (4.1 ± 0.4 fold), an effect that was almost additive to *ANGPTL3*-siRNA. Indeed, the combination of human recombinant ANGPTL3 and *ANGPTL3*-siRNA determined a maximum effect on intracellular lipid content (8.6 ± 0.2 fold, Figure 1D,E). Thus, our experimental condition very closely resembled the effect of vupanorsen on lipid liver accumulation [11,12]. 

**Figure 2:**
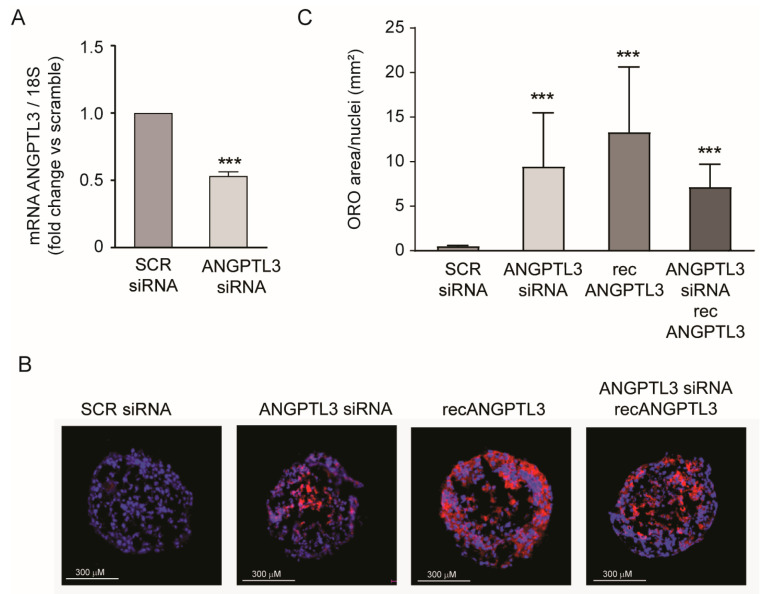
*ANGPTL3*-siRNA induced lipid accumulation in Huh7-LX2 spheroids. (**A**) RT-qPCR was performed on spheroids, and data expressed as ΔΔCt referred to cells transfected with siRNA-scramble. (**B**) Representative images of intracellular neutral lipid content visualized by Oil Red-O staining. Blue is DAPI (nuclei), red is Oil Red-O (neutral lipids). (**C**) Histograms of quantification of Oil Red-O stained area relative to nuclei was performed with ImageJ. All data are presented as mean ± SD of three independent experiments. Representative images for each experiment were chosen. *p* value was calculated using Student’s *t*-test. *** *p* < 0.001 vs. siRNA-scramble.

To further corroborate our findings, we utilized a similar approach in Huh7-LX2 (24:1) spheroids. *ANGPTL3*-siRNA was reduced by 46.9% compared with *ANGPTL3* mRNA expression (Figure 2A). This effect was sufficient to strongly induce lipid accumulation, determined by Oil Red-O staining (Figure 2B,C). Similarly to what was observed in the Huh7 cell line, human recombinant ANGPTL3 also induced intracellular lipid accumulation, although with higher variability. The effect of lipid accumulation of *ANGPTL3*-siRNA was also confirmed in the combination with 100 ng/mL of human recombinant ANGPTL3 (Figure 2B,C).

**Figure 3:**
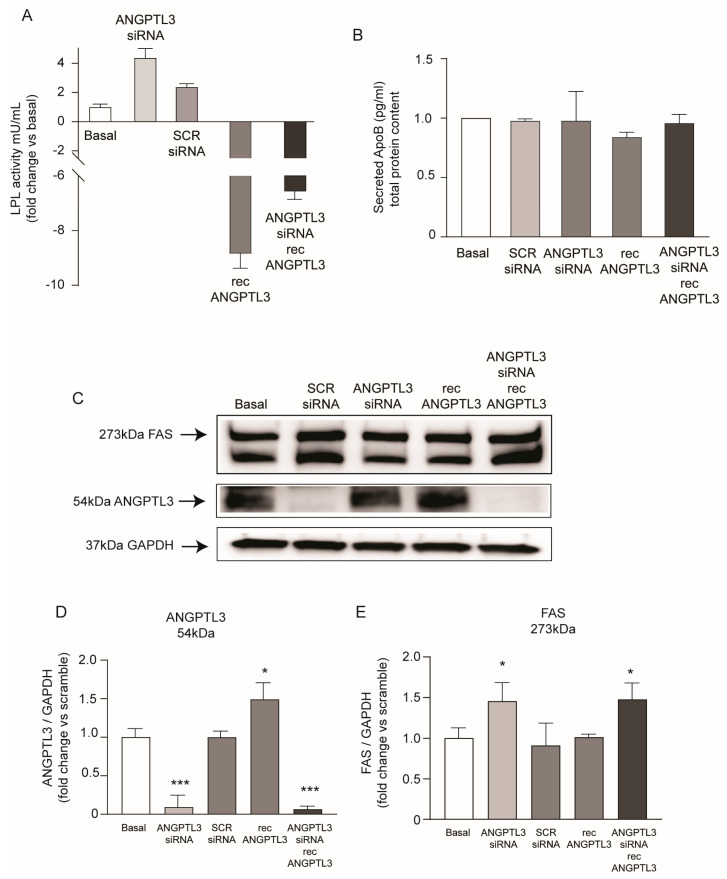
*ANGPTL3*-siRNA induced FAS in Huh7 cell line. (**A**) Cells were transfected with scramble-siRNA and *ANGPTL3*-siRNA, and incubated for 24 h, then recANGPTL3 was added. Then, 24 h later, LPL assay was performed, and obtained data are expressed as milliunits per liter vs. basal condition. (**B**) ELISA on secreted apoB was performed on cell supernatant after 48 h of incubation. Data are expressed as fold change vs. basal after normalization on total protein content. (**C**) ANGPTL3 and FAS expression were determined by Western blot analysis and GAPDH was used as the loading control. (**D**,**E**) Histograms of quantification of Western blot analysis. All data are presented as mean ± SD of three independent experiments. *p* value was calculated using Student’s *t*-test. * *p* < 0.05, *** *p* < 0.001 vs. basal.

Considering the physiological role of ANGPTL3, we determined the LPL activity from the total cell lysates of the Huh7 cell line (Figure 3A). We detected a very low basal activity of LPL which significantly increased by 4.4-fold after silencing of *ANGPTL3* and was inhibited by the addition of recombinant ANGPTL3. A lower inhibitory effect on LPL activity was observed in Huh7 cells incubated with the combination of *ANGPTL3*-siRNA and recANGPTL3. No significant changes were detected in apoB concentrations in the conditioned media, indicating that genetic manipulation of ANGPTL3 did not modulate the production of apoB-containing lipoprotein (Figure 3B). On the contrary, a significant increase in FAS was detected by total protein lysates of Huh7 cells in response to *ANGPTL3*-siRNA (1.45-fold). A similar effect was observed in the presence of human recombinant ANGPTL3 (1.47-fold). These results indicated that the lipid accumulation observed after gene silencing of *ANGPTL3* is stimulated by the activation of the de novo lipogenesis that was not associated with increased secretion of apoB-containing lipoproteins.

**Figure 4:**
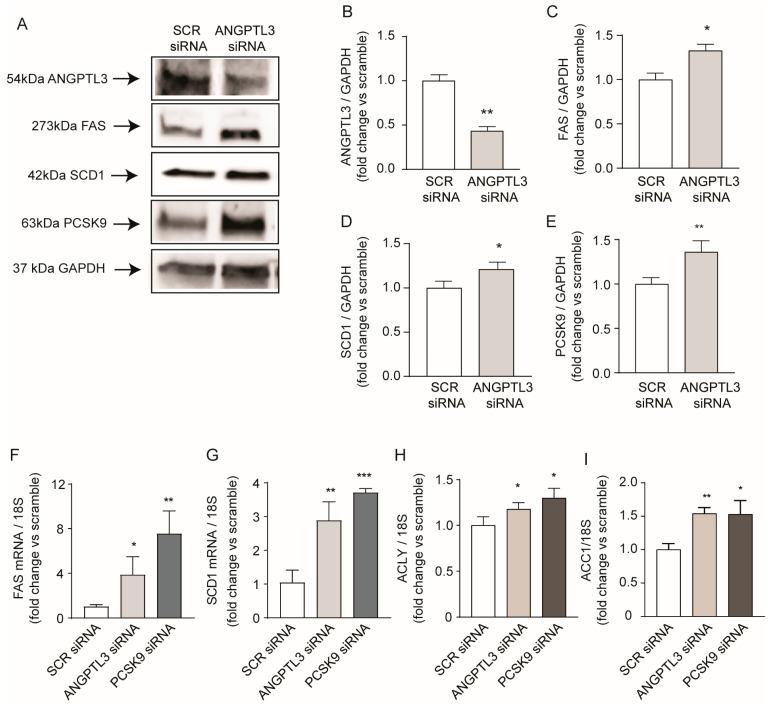
*ANGPTL3*-siRNA induced genes regulated by SREBP1 transcription factor. (**A**) Cells were transfected with scramble-siRNA or *ANGPTL3*-siRNA and incubated for 48 h. ANGPTL3, FAS, SCD1, and PCSK9 expression were determined by Western blot analysis and GAPDH was used as the loading control. (**B**–**E**) Histogram graphs show the relative protein amount calculated as protein/GAPDH vs. siRNA-scramble. (**F**–**I**) Cells were transfected with scramble-siRNA, *PCSK9*-siRNA, or *ANGPTL3*-siRNA and incubated for 48 h. mRNA levels of *FAS*, *SCD1*, *ACLY*, and *ACC1* were determined by quantitative real-time PCR. All data are presented as mean ± SD of three independent experiments. *p* value was calculated using Student’s *t*-test. * *p* < 0.05, ** *p* < 0.01, *** *p* < 0.001, vs. siRNA-scramble.

A more complete analysis of proteins involved in lipid metabolism revealed that, together with the induction of FAS (1.33-fold), SCD1 was also induced by 1.21-fold in response to gene silencing of *ANGPTL3* (Figure 4A,C,D). More intriguingly, we observed a significant induction of PCSK9 expression, suggesting the activation of the sterol regulatory element binding protein 1 (SREBP1) transcription factor that modulates all these genes [24,25]. Indeed, mRNA levels of *FAS*, *SCD1*, ATP citrate lyase (*ACLY*), and Acetyl-Coenzyme A Carboxylase 1 (*ACC1*) genes that are transcriptionally regulated by *SREBP1* were induced after silencing of *ANGPTL3* (3.8-fold, 2.9-fold, 1.2-fold, and 1.5-fold for *FAS*, *SCD1*, *ACLY*, and *ACC1*, respectively; Figure 4F–I).

Since we observed that PCSK9 is induced in response to *ANGPTL3* silencing, we decided to study the effect of *PCSK9*-siRNA on the expression of lipid-related genes. As shown in Figure 4, *PCSK9* silencing showed an even stronger induction of de novo lipogenesis genes compared to *ANGPTL3*-siRNA with a 7.5-fold, 3.7-fold, 1.3-fold, and 1.5-fold increase in mRNA levels of *FAS*, *SCD1*, *ACLY*, and *ACC1*, respectively (Figure 4F–I). This evidence opens the possibility that PCSK9 and ANGPTL3 may be mutually regulated and that both could contribute to the final regulation of intracellular lipid homeostasis.

**Figure 5:**
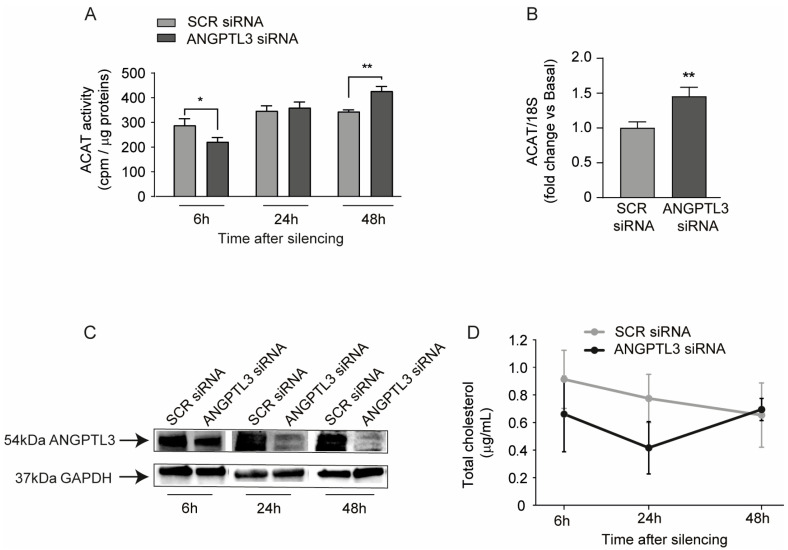
Effect of *ANGPTL3*-siRNA on ACAT activity. (**A**,**B**) Cells were transfected with scramble-siRNA and *ANGPTL3*-siRNA and incubated for an additional 6, 24, and 48 h. (**A**) Cholesterol esterification was measured by incubating cells with [1-^14^C]oleic acid for 4 h. (**B**) mRNA levels of *ACAT* were determined by quantitative real-time PCR. (**C**) ANGPTL3 expression was determined by Western blot analysis and GAPDH was used as the loading control. (**D**) Total cholesterol determination by LC-APCI-MS. *p* value was calculated using one-way ANOVA. * *p* < 0.05; ** *p* < 0.01 vs. scramble-siRNA.

The SREBP pathway is induced in response to free intracellular sterol deprivation [26]. We thus determined the Acyl-coenzyme A cholesterol acyltransferase (ACAT) activity at different time points post transfection with *ANGPTL3*-siRNA. After 6, 24, and 48 h of ANGPT3 gene silencing, Huh7 cells were incubated with [1-^14^C]oleic acid for 4 h and its incorporation into cholesterol esters was determined. Interestingly, we observed a partial reduction in ACAT activity 6 h post silencing and a significant induction at 48 h (Figure 5A). These data indicated that the silencing of intracellular ANGPTL3 determined, at early time points, a partial deprivation of free cholesterol as a substrate of ACAT enzyme, a condition that is restored at 24 h and with the increase in cholesterol-ester observed at 48 h. Indeed, the determination of total cholesterol levels indicated a trend of intracellular reduction in response to *ANGPTL3*-siRNA at early time points (6 h and 24 h) (Figure 4D). *ACAT* mRNA was also induced at 48 h, consistently with the higher cholesterol esterification in response to *ANGPTL3* silencing (Figure 4B). Western blot analysis confirmed that *ANGPTL3*-siRNA efficiently affected its expression at as early as 6 h, with a stronger effect at longer time points (Figure 4C).

**Figure 6:**
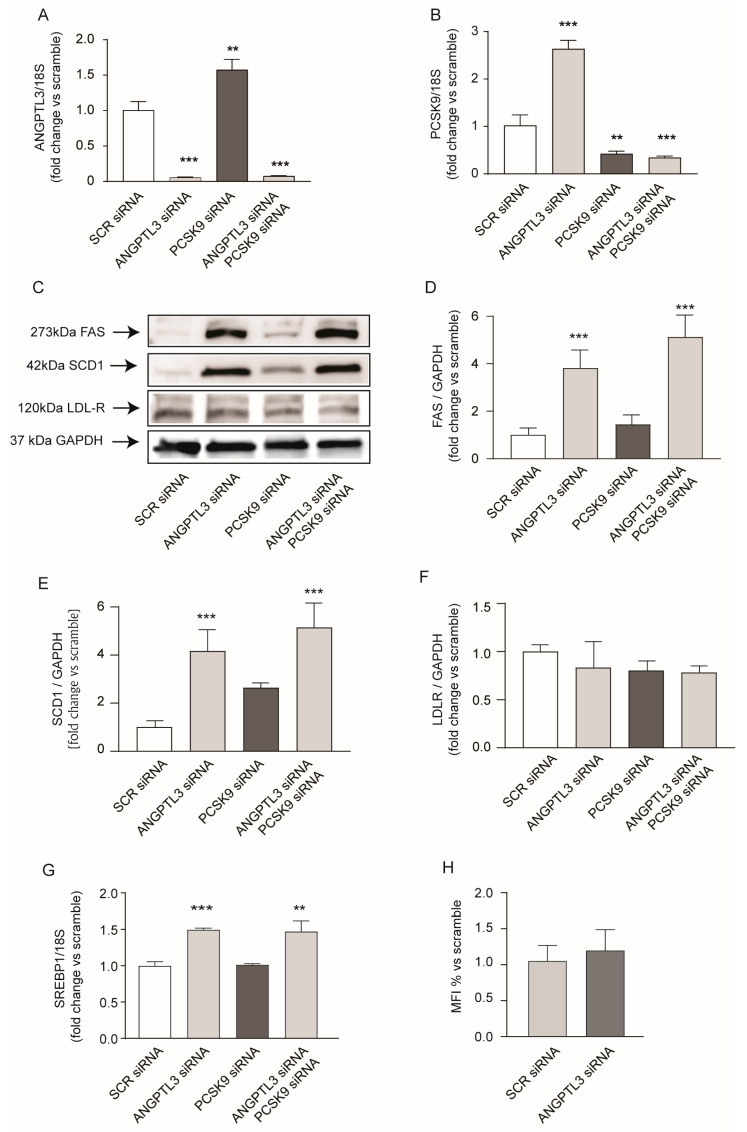
*ANGPTL3*-siRNA induced genes involved in the de novo lipogenesis independently from PCSK9. (**C**–**F**) Cells were transfected with scramble-siRNA, *ANGPTL3*-siRNA, or *PCSK9*-siRNA and incubated for 48 h. FAS, SCD1, and LDL-R expression were determined by Western blot analysis and GAPDH was used as the loading control (**C**). Histogram shows the relative protein amount calculated as protein/GAPDH vs. scramble-siRNA (**D**–**F**). (**A**,**B**,**G**) mRNA levels of *ANGPTL3*, *PCSK9*, and *SREBP1* were determined by quantitative real-time PCR 48 h after treatment. (**H**) Under the same experimental conditions described for panel (**C**–**F**) the LDL-DyLight^TM^ 550 uptake was determined by flow cytometry and indicated as mean fluorescence index (MFI%). All data are presented as mean ± SD of three independent experiments. *p* value was calculated using Student’s *t*-test. ** *p* < 0.01, *** *p* < 0.001 vs. siRNA-scramble.

PCSK9 has been directly associated with plasma TG levels [27] and liver fat accumulation [28]; therefore, we investigated its possible involvement in lipid accumulation in response to *ANGPTL3*-siRNA. To test this hypothesis, we performed a double gene silencing by co-transfecting Huh7 with siRNA targeting either *ANGPTL3* or *PCSK9*. Both genes were significantly downregulated after single or double siRNA transfection (Figure 6A,B). More interestingly, we observed a mutual regulation of *PCSK9* and *ANGPTL3*; indeed, after transfection with *ANGPTL3*-siRNA, *PCSK9* mRNA was upregulated (Figure 6B) and vice versa, gene silencing of *PCSK9* induced *ANGPTL3* mRNA levels (Figure 6A).

As previously observed, the absence of ANGPTL3 significantly induced FAS and SCD1, and this effect was also superimposable after gene silencing of *PCSK9* (Figure 6C–E). PCSK9 silencing showed a minor inducing effect on SCD1 without changing the expression of the LDL receptor (Figure 6F), and *ANGPTL3*-siRNA did not change either the LDL receptor expression or LDL DyLight^TM^ 550 uptake (Figure 6F,G). Thus, *ANGPTL3*-siRNA induced genes involved in the de novo lipogenesis independently from PCSK9, most likely by activating the *SREBP1* transcription factor, as observed by quantitative real-time PCR analysis (Figure 6G).

**Figure 7:**
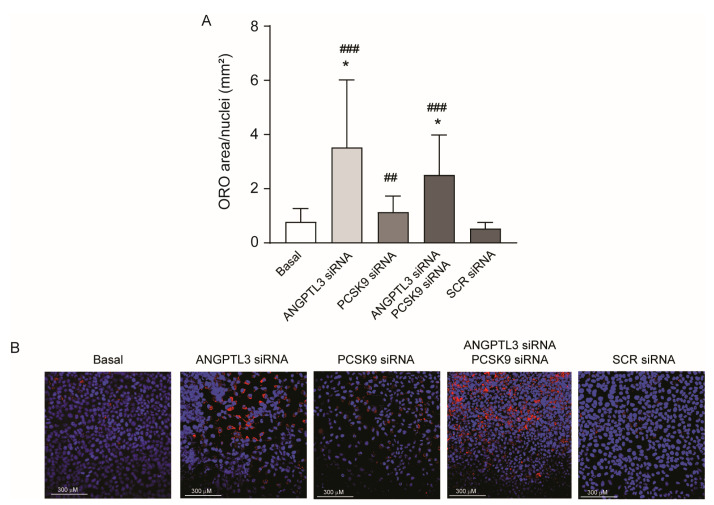
*ANGPTL3*-siRNA induced lipid accumulation in human hepatoma cell line Huh7 independently from PCSK9. (**A**,**B**) Cells were transfected with siRNA-scramble, *ANGPTL3*-siRNA, or *PCSK9*-siRNA, and incubated for 48 h. Intracellular neutral lipid content was visualized by Oil Red-O staining. (**A**) Quantification of Oil Red-O area relative to nuclei was performed with ImageJ v. 1.54d. (**B**) Representative images, blue is DAPI (nuclei), red is Oil Red-O (neutral lipids). All data are presented as mean ± SD of three independent experiments. *p* value was calculated using Student’s *t*-test. * *p* < 0.05, vs. siRNA-scramble. ^##^ *p* < 0.01, ^###^ *p* < 0.001 vs. basal.

Finally, Oil Red-O staining confirmed that lipid accumulation in response to *ANGPTL3*-siRNA also occurred when co-transfected with *PCSK9*-siRNA (Figure 7A,B).

Taken together, the absence of ANGPTL3 determined lipid accumulation in human hepatoma cell line Huh7, by promoting the de novo lipogenesis independently from PCSK9, and without determining an increase in the secretion of apoB-containing lipoproteins.

## 3. Discussion

In the present study, we investigated the basic molecular mechanisms by which the treatment with oligonucleotide antisense (ASO) vupanorsen could have determined the dose-dependent increase in hepatic fat seen in human trials [18]. We first confirmed this side effect in vitro by using two different approaches, both human hepatoma cell line Huh7 in 2D, and a 3D system that resembles the liver more closely, made of Huh7-LX2 spheroids. The lipid accumulation was observed after silencing *ANGPTL3* and in the presence of exogenous human recombinant ANGPTL3 at concentrations similar to those observed in human plasma (100 ng/mL) [23]. Regarding the interaction of ANGPTL3 with hepatocytes, a specific mechanism is currently unknown. The most plausible hypotheses explaining the observed effect are provided by Ruhanen et al. and suggest the potential interaction of ANGPTL3 with liver cells through its fibrinogen-like domain, stimulating the PI3K/Akt/mTOR pathway, or by endocytosis to execute intracellular functions within endosomes [29]. Under our experimental conditions, targeting gene expression of *ANGPTL3* caused a significant induction of key enzymes involved in the de novo lipogenesis, such as FAS, SCD1, ACLY, and ACC1, without any changes in the extracellular levels of apoB. These data indicated that gene silencing of *ANGPTL3* induced lipid accumulation by increasing their synthesis. The lack of effect on apoB secretion is in line with a previous study conducted in mice [14]. However, more recent in vitro study conducted with CRISPR-associated protein 9 (CRISPR/Cas9) to target *ANGPTL3* in HepG2 cells (ANGPTL3^-/-^) showed a 50% reduction in apoB100 secretion, associated with its early presecretory degradation [30]. Using this approach, the knock-down of ANGPTL3 did not result in neutral lipid accumulation, most likely due to increased fatty acid oxidation. The discrepancy between these and our findings can be attributed to the experimental approach utilized, such as partial gene silencing with siRNA vs. total knock-out with CRISPR/Cas9. In addition, our experiments were performed in absence of oleic acid, while the decreased secretion of ApoB100 in ANGPTL3^-/-^ was observed in oleic acid-containing cell culture media [30]. Similarly to our study, Xu et al. also observed neutral lipid accumulation in Huh7 cells [31]. The differences between these studies can potentially be explained by considering that HepG2 cells, utilized by Burks et al., seem to lipidate ApoB100 more efficiently in the presence of oleate, compared to Huh7 [30]. Thus, the absence of oleic acid limits the secretion of ApoB-containing lipoproteins, determining lipid accumulation in response to *ANGPTL3* gene silencing. Finally, the different results could be explained by distinct metabolic activity between the two cell lines.

*FAS* and *SCD1* are transcriptionally regulated by SREBP1, thus suggesting that the absence of ANGPTL3 may interfere with the SREBP cleavage-activating protein (SCAP), a protein that contains a sterol-sensing domain. In cholesterol-depleted cells, this protein binds to SREBP1 and mediates its transport from the endoplasmic reticulum (ER) to the Golgi apparatus, where it undergoes proteolytical cleavage and stimulates sterol biosynthesis [32,33]. Gene silencing of *ANGPTL3* was associated with a higher activity of LPL measured from total protein extracts, thus suggesting a possible change in the ratio of free and esterified cholesterol. Thus, it is conceivable to hypothesize that the absence of ANGPTL3 may reduce the intracellular concentration of free cholesterol, determining the activation of the SREBP pathway. Indeed, by measuring the ACAT activity, we observed that after 6 h of transfection with *ANGPTL3*-siRNA, the amount of cholesterol esterification was reduced, which could be a direct consequence of lower free cholesterol as a substrate. In agreement with these data, we also observed a downward trend of total intracellular cholesterol levels at 6 and 24 h with a complete recovery at 48 h. In a previous study by Ruhanen et al., cholesterol esters were markedly reduced in *ANGPTL3* knock-down by CRISPR/Cas9 cells, and *ACAT* mRNA was reduced, as we observed at 6 h post treatment [34]. This could lead to a new consideration, which is the timing of the experiments. As observed in our kinetic experiment (Figure 4), we observe an opposite result between 6 and 48 h after silencing. This could be partially explained as an initial effect of metabolic shift due to the silencing itself, followed then, at 48 h, by a shift towards other pathways that results in a cumulative pro-lipid accumulation effect.

A second relevant finding of our study is that in response to gene silencing of *ANGPTL3*, a significant increase in PCSK9 expression was observed, most likely related to the activation of the SREBP pathway [35]. PCSK9 is a well-known regulator of the LDL receptor, and clinical data firmly determined a positive association between its plasma levels and TG concentration [27], and liver fat accumulation [28]. PCSK9 has been shown to directly interact with apoB in hepatocytes and to drive their mutual secretion into the circulation [36]. However, under our experimental conditions, we did not find a significant increase in apoB from cultured hepatocytes, or in PCSK9 with their intracellular accumulation. In this regard, a direct interaction between ANGPTL3 and PCSK9 has been documented [37]. Whether this interaction may alter the PCSK9-dependent apoB secretory pathway is still unknown. Being aware of this potential interaction between ANGPTL3 and PCSK9, and having observed that the expression of these two proteins was inversely regulated, we conducted double silencing experiments to determine whether the effect could be mutual or driven by one of them. Not observing a rescue of activity with double silencing, but witnessing a behavior comparable to the sole silencing of *ANGPTL3*, we demonstrated that intracellular lipid accumulation in response to *ANGPTL3* gene silencing is independent of the presence of PCSK9. However, the two proteins might contribute differently to the ultimate accumulation. Furthermore, we observed that the accumulation mechanism may not be attributed to an increased uptake of LDL cholesterol particles, as evidenced by the absence of alterations not only in LDLR protein expression but also in the uptake of fluorescent LDL. Therefore, we excluded involvement of the LDL receptor in the lipid accumulation mechanism, but certainly, other uptake pathways could be considered in future investigations. Certainly, with the growing body of evidence, it is clear that the de novo lipogenesis pathway may not be the only pathway involved in lipid accumulation resulting from *ANGPTL3* silencing; nevertheless, it constitutes a significant portion of it. Previous experiments have already noted altered expression of lipid metabolism-related pathways in ANGPTL3 knock-down (KD) cells [34]. Conversely, the absence of ANGPTL3 from birth does not appear to have any detrimental cardiometabolic effects; in fact, it seems to be protective [34]. Regarding this, the study of potential compensatory mechanisms in individuals lacking ANGPTL3 from birth, without experiencing hepato-related side effects, would certainly be intriguing.

## 4. Materials and Methods

### 4.1. Cell Cultures

Cell culture reagents and plastic supply were purchased from EuroClone (Milan, Italy) if not otherwise specified. Huh-7 cell line was maintained in Modified Eagle’s Medium (MEM) supplemented with 10% Fetal Bovine Serum (FBS), 1% penicillin/streptomycin solution (10,000 U/mL and 10 mg/mL, respectively), 1% L-glutamine 200 mM, and 1% non-essential amino acids 100× solution. LX-2 cell line was maintained in Dulbecco Modified Eagle’s Medium (DMEM) supplemented with 10% Fetal Bovine Serum (FBS), 1% penicillin/streptomycin solution (10,000 U/mL and 10 mg/mL, respectively), and 1% L-glutamine 200 mM. Spheroids were made mixing Huh7-LX2 cells in 24:1 ratio at 2000 cells/well density using BIOFLOAT cell culture plates by Sarstedt (cod. 83.3925.400) and maintained for 96 h.

### 4.2. ANGPTL3 Silencing and Recombinant Protein Administration

Cells were seeded and then grown to 70% confluence in MEM/10% FBS. Cells were washed with PBS (SIGMA-Aldrich, St. Louis, MO, USA) and fresh culture medium was added to each plate and then transfected with a validated ANGPTL3-siRNA (Cat# AM16708) or negative control scramble-siRNA purchased from Thermo-Scientific and mixed to obtain a 50 mM stock solution in nuclease free water provided by the supplier. Silencing was performed with Lipofectamine™ 3000 Transfection Reagent (Thermo-Scientific, Waltham, MA, USA, Catalog number: L3000001) according to manufacturer’s instructions. Cells were incubated for 48 h and then treated accordingly for further experiments. To detect silencing efficiency, RT-qPCR was performed according to the method described in the RT-qPCR section. Human Recombinant Angiopoietin-like 3 with C-terminal flag tag was purchased by BPS Bioscience^®^ (San Diego, CA, USA) and reconstituted as stated in the datasheet. The recombinant protein was always added to the cells 24 h after silencing.

### 4.3. Western Blotting

A total of 200,000 cells/well were seeded and treated 24 h later according to the experiment. After 48 h, cells were washed twice with PBS (SIGMA-Aldrich) and homogenized in lysis buffer containing 1% NP-40, 150 mM NaCl, and 50 mM Tris-HCl at pH 7.5. Protein concentration was assessed by BCA assays (Euroclone), according to manufacturer’s instructions. The 25 μg total protein extract/samples were separated on 4–20% SDS-Page gel (Bio-Rad, San Francisco, CA, USA) under denaturing and reducing conditions. Proteins were then transferred onto a nitrocellulose membrane by using the Trans-Blot^®^ Turbo™ Transfer System (Bio-Rad); 5% non-fat dried milk in tris-buffered saline containing 0.2% of tween 20 (TBST20) was used as blocking buffer. All the primary antibodies were diluted in 5% non-fat dried milk in TBST20 and incubated overnight at 4 °C in agitation. Horseradish peroxidase (HPR) conjugated secondary antibodies were diluted in blocking solution and membranes were left to incubate 90 min at room temperature (RT) in agitation. Luminescence signals were acquired with Uvitec Alliance Q9 (Uvitec, Cambridge, UK). Quantitative densitometric analysis was performed with FIJI ImageJ free software v1.54d. When used, stripping buffer was prepared according to Abcam’s recipe. PCSK9 antibody was from GeneTex (Irvine, CA, USA) (cod. GTX129859; dilution 1:1000), ANGPTL3 antibody was from GeneTex (cod GTX104569; dilution 1:1000), FAS antibody was from Abclonal (cod. A21182; dilution 1:1000), SREBP2 antibody was from Abcam (Waltham, MA, USA) (cod ab30682; dilution 1:1000), LDLR antibody was from GeneTex (cod. GTX37639; dilution 1:1000) GAPDH antibody was from GeneTex (cod. GTX100118; dilution 1:5000), SCD1 antibody was from AbClonal (Woburn, MA, USA) (cod. A16429, dilution 1:1000), and anti-rabbit secondary antibody was from Jackson ImmunoResearch (Cambridge, UK) (cod. 113-036-045, dilution 1:5000).

### 4.4. Reverse Transcription and Quantitative PCR (RT-qPCR)

Total RNA was extracted using the iScript™ RT-qPCR Sample Prep reagent (Bio-Rad), according to the manufacturer’s instructions. QuantiNova SYBR Green RT-PCR Kit (QIAGEN, Hilden, Germany) was used for qPCR, along with specific primers for 18S (FWD 5′-CGGCTACCACATCCACGGAA-3′, REV 5′-CCTGAATTGTTATTTTTCGTCACTACC-3′) PCSK9 (FWD 5′-CCTGCGCGTGCTCAACT-3′, REV 5′-GCTGGCTTTTCCGAATAAACTC-3′), ANGPTL3 (FWD 5′-GCCTGTTGGAGACTCAGATGG-3′, REV 5′-TAGCACCTTCTGTGCCTGGG-3′), FAS (FWD 5′-GCAAATTCGACCTTTCTCA-3′, REV 5′-GGACCCCGTGGAATGTCA-3′), ACLY (FWD 5′-TGCAAAGTGAAGTGGGGTGA-3′, REV 5′-TTTGGGGTTCAGCAAGGTCA-3′), ACC1 (FWD 5′-ATGTCTGGCTTGCACCTAGTA-3′, REV 5′-CCCCAAAGCGAGTAACAAATTCT-3′), PCSK9 (FWD 5′-CCTGCGCGTGCTCAACT-3′, REV 5′-GCTGGCTTTTCCGAATAAACTC-3′), SCD1 (FWD 5′-AAAGCGAGGTGGCCATGTTA-3′, REV 5′-TCATGCCTCAAAACTGCCCT-3′).

The analyses were performed with the CFX96 Touch Real-Time PCR Detection System (Bio-Rad) with cycling conditions of 45 °C for 10 min, 95 °C for 5 min, and a repetition of 40 cycles at 95 °C for 5 s followed by 30 s at 60 °C. The data were expressed as Ct values and used for relative quantification of targets with ΔΔCt calculations. The ΔΔCt values were determined by multiplying the ratio value between the efficiency of specific primers and housekeeping 18S. The efficiency was calculated as ((10^(−1/slope)^) − 1) × 100.

### 4.5. ELISA Assay for ApoB and PCSK9

To detect the secreted amount of ApoB and PCSK9, the Human ApoB ELISA kit (Fine Test, Wuhan, China, cod. EH0620) and human PCSK9 ELISA kit (R&D Systems, Inc., Minneapolis, MN, USA, 614 McKinley Place NE, cod. DY3888) were used according to the manufacturer’s instructions. Cells were seeded in 6 well plates (200,000 cells/well) in MEM/10% FBS, and 24 h later the treatments were performed. After 48 h the supernatant was collected, centrifuged at 15,000 rpm for 10 min and diluted according to the manufacturer’s instructions. Absorbance at 450 nm was obtained with VICTOR Nivo Multimode Microplate Reader (PerkinElmer, Waltham, MA, USA).

### 4.6. Neutral Lipid Staining with Oil Red-O

To perform Oil Red-O staining, Huh7 cells were seeded 40,000 cells/well in 24-well plates with sterile microscope cover glasses 10 mm Ø (VWR international, Atlanta, GA, USA). After 24 h the medium was replaced by fresh MEM/10% FBS containing the described treatments. Then, 48 h later, cells were rinsed with PBS and fixed in paraformaldehyde (PFA, Sigma-Aldrich). Neutral lipid content was measured using Oil Red-O staining technique as previously stated [38]. Oil Red-O powder was purchased from Sigma-Aldrich (Cod. O0625). Nuclei were stained with DAPI solution in PBS (Sigma-Aldrich, cod. D9542). The same staining protocol was followed for spheroids. Three-dimensional spheroids were fixed with 10% paraformaldehyde (PFA, Sigma-Aldrich) for 2 h, after washing with PBS, they were incubated with 20% *w*/*v* sucrose (Sigma-Aldrich, cod. D9542) in PBS overnight. After washing 3 times with PBS, the spheroids were embedded in OCT and stored at −80 °C. Spheroids were sectioned into 8 μm-thick slices using cryostat and the sections were stored at −80 °C until staining. The staining procedure is the same as with the 2D cell cultures. The slides or the coverslips were mounted with Fluoromount™ Aqueous Mounting Medium (Sigma-Aldrich, Cod. F4680). Images were obtained with Leica DMRE mounting Leica camera with Leica 541 517 HC zoom and Leica Application Suite X Software (Las X v.3.7.6). Oil Red-O stained areas were quantified using ImageJ (v.1.52h, NIH, Bethesda, MD, USA) and normalized with nuclei count.

### 4.7. Lipoprotein Lipase Activity Assay

Lipoprotein Lipase activity assay kit (Abcam, cod. ab204721) was used to assess the performance of the enzyme after the treatments. The protocol was followed according to the manufacturer’s instructions.

### 4.8. Cholesterol Esterification Assay (ACAT Activity)

ACAT activity was performed as previously described [39]. In brief, Huh7 cells were seeded in 24-well plates in MEM supplemented with 10% FCS (both from Euroclone, Milan, Italy) at a density of 30,000 cells/well and allowed to adhere for 24 h. After gene silencing previously described, cholesterol esterification was measured by incubating cells with [1-^14^C]oleic acid (0.85 µCi/sample; Perkin Elmer, Waltham, MA, USA) complexed with fatty acid-free bovine serum albumin (BSA; Merck, Darmstadt, Germany) for 4 h. At the end of the incubation period, cells were washed twice with ice-cold phosphate-buffered saline (PBS) and a fixed amount of [^3^H]oleic acid (0.005 µCi/sample; Perkin Elmer, Waltham, MA, USA) was added to each sample as an internal standard. Cellular lipids were extracted by incubating monolayers with a mixture of hexane/isopropanol (3:2) for 30 min with gentle shaking. The extracted lipids were separated by thin layer chromatography (TLC) using a mixture of isooctane/diethyl ether/acetic acid (75:25:2, *v*/*v*/*v*) as mobile phase. Esterified cholesterol radioactivity in each spot was quantified by liquid scintillation counting (Perkin Elmer, Waltham, MA, USA). Data were expressed as cpm of [1-^14^C]oleic acid corrected per microgram of protein of each cell lysate measured by the BCA assay according to the manufacturer’s instructions.

### 4.9. Cholesterol Determination

Cell monolayers were washed with PBS (phosphate buffer saline) and incubated for 2 h at RT with 0.1 M NaOH. The total cholesterol content of cells was measured using liquid chromatography coupled with mass spectrometry with atmospheric pressure chemical ionization ion source (LC-APCI-MS). The system used for analysis was an Agilent 1260 Liquid chromatograph, coupled with a Varian mass spectrometer MS 500 with ion trap analyzer. For the chromatographic separation, an Agilent XDB C-18 3.0 × 150 mm was used. Elution was performed using a mixture of acetonitrile 87%, Methanol 10%, water 0.1%, and formic acid 3%, in isocratic mode for 15 min. Spectra were acquired in the range *m*/*z* 350–550. Cholesterol was detected as [M-H_2_O+H]^+^ at *m*/*z* 369.5. A cholesterol calibration curve was created in the range 50.0–0.5 µg/mL. Samples were prepared as follows: a liquid/liquid partition was performed adding chloroform to lysates. Samples were dried and then diluted with equal volume of chloroform and finally used for chromatography.

### 4.10. Fluorescent LDL Uptake Cell-Based Assay

Huh7 cells were seeded in 6-well tray (3 × 10^5^ cells/well in a complete medium) and after 24 h, treated in MEM/0.4% FBS media. A total of 24 h after treatment, cells were washed with PBS and incubated with 10 µg/mL of LDL-DyLight^TM^ 550 (Cayman Chemicals cod. 10011229) in 0.4% FCS media. After 3 h of incubation at 37 °C, cells were washed with PBS, detached with trypsin, and resuspended in MEM/10% FBS. After centrifugation (4 min at 3500 rpm), the pellet is resuspended in PBS and each sample was transferred to a cytofluorometer tube. The fluorescence was measured by using a flow cytometer (BD FACSAria™ III, DB Life Sciences, San Jose, CA, 95131, USA) at excitation and emission wavelength of 484 nm.

### 4.11. Statistical Analysis

Data are expressed as mean ± standard deviation. To compare differences between two conditions, *p* values were determined by Student’s *t*-test using GraphPad^®^ Software v8.2.1 for Windows. Otherwise, differences between treatment groups were evaluated by one-way ANOVA. A probability value of *p* < 0.05 was considered statistically significant. If not stated, *p* value was above 0.05.

## 5. Conclusions

From our in vitro data, it is possible to conclude that the activation of the de novo lipogenesis represents one possible mechanism by which the vupanorsen determined the hepatic fat accumulation. This effect was observed either in the absence or in the presence of exogenous human recombinantANGPTL3, indicating a direct role of intracellular ANGPTL3 on lipid homeostasis. Indeed, the treatment of mAb evinacumab, which blocks the exogenous ANGPTL3, seems to determine a lipid lowering effect without significant changes in hepatic fat content [9,10]. Given that monoclonal antibodies (mAb) target the circulating protein while antisense oligonucleotides (ASO) target the hepatic pool, the clinical results lead us to believe that there are still intrahepatic roles of ANGPTL3 that need to be elucidated. This work contributes to affirming that the regulation of intrahepatic lipid metabolism may be one of these roles. The de novo lipogenesis is most likely induced by reducing the intracellular free-cholesterol content. The alteration in the ratio between free and esterified cholesterol can be a result of the activation of lipolysis, which may increase the availability of intracellular free fatty acids, which in turn, may be available for cholesterol esterification by ACAT enzyme. In line with our observation, reduced lipid content, associated with the activation of SREBP pathway, has been recently documented in regulatory T cells isolated from patients affected by familial combined hypolipidemia type 2 (ANGPTL3 deficiency) [40]. Nevertheless, additional analyses are required to address this hypothesis as well as the possible clinical significance of the co-regulation between ANGPTL3 and PCSK9.

## Data Availability

Data contained within the article.

## References

[1] Conklin D., Gilbertson D., Taft D.W., Maurer M.F., Whitmore T.E., Smith D.L., Walker K.M., Chen L.H., Wattler S., Nehls M. (1999). Identification of a mammalian angiopoietin-related protein expressed specifically in liver. Genomics.

[2] Koishi R., Ando Y., Ono M., Shimamura M., Yasumo H., Fujiwara T., Horikoshi H., Furukawa H. (2002). Angptl3 regulates lipid metabolism in mice. Nat. Genet..

[3] Shimizugawa T., Ono M., Shimamura M., Yoshida K., Ando Y., Koishi R., Ueda K., Inaba T., Minekura H., Kohama T. (2002). ANGPTL3 decreases very low density lipoprotein triglyceride clearance by inhibition of lipoprotein lipase. J. Biol. Chem..

[4] Shimamura M., Matsuda M., Kobayashi S., Ando Y., Ono M., Koishi R., Furukawa H., Makishima M., Shimomura I. (2003). Angiopoietin-like protein 3, a hepatic secretory factor, activates lipolysis in adipocytes. Biochem. Biophys. Res. Commun..

[5] Shimamura M., Matsuda M., Yasumo H., Okazaki M., Fujimoto K., Kono K., Shimizugawa T., Ando Y., Koishi R., Kohama T. (2007). Angiopoietin-like protein3 regulates plasma HDL cholesterol through suppression of endothelial lipase. Arterioscler. Thromb. Vasc. Biol..

[6] McCoy M.G., Sun G.S., Marchadier D., Maugeais C., Glick J.M., Rader D.J. (2002). Characterization of the lipolytic activity of endothelial lipase. J. Lipid Res..

[7] Lupo M.G., Ferri N. (2018). Angiopoietin-Like 3 (ANGPTL3) and Atherosclerosis: Lipid and Non-Lipid Related Effects. J. Cardiovasc. Dev. Dis..

[8] Dewey F.E., Gusarova V., Dunbar R.L., O’Dushlaine C., Schurmann C., Gottesman O., McCarthy S., Van Hout C.V., Bruse S., Dansky H.M. (2017). Genetic and Pharmacologic Inactivation of ANGPTL3 and Cardiovascular Disease. N. Engl. J. Med..

[9] Ruscica M., Zimetti F., Adorni M.P., Sirtori C.R., Lupo M.G., Ferri N. (2020). Pharmacological aspects of ANGPTL3 and ANGPTL4 inhibitors: New therapeutic approaches for the treatment of atherogenic dyslipidemia. Pharmacol. Res..

[10] Gusarova V., Alexa C.A., Wang Y., Rafique A., Kim J.H., Buckler D., Mintah I.J., Shihanian L.M., Cohen J.C., Hobbs H.H. (2015). ANGPTL3 blockade with a human monoclonal antibody reduces plasma lipids in dyslipidemic mice and monkeys. J. Lipid Res..

[11] Gaudet D., Karwatowska-Prokopczuk E., Baum S.J., Hurh E., Kingsbury J., Bartlett V.J., Figueroa A.L., Piscitelli P., Singleton W., Witztum J.L. (2020). Vupanorsen, an N-acetyl galactosamine-conjugated antisense drug to ANGPTL3 mRNA, lowers triglycerides and atherogenic lipoproteins in patients with diabetes, hepatic steatosis, and hypertriglyceridaemia. Eur. Heart J..

[12] Graham M.J., Lee R.G., Brandt T.A., Tai L.J., Fu W., Peralta R., Yu R., Hurh E., Paz E., McEvoy B.W. (2017). Cardiovascular and Metabolic Effects of ANGPTL3 Antisense Oligonucleotides. N. Engl. J. Med..

[13] Raal F.J., Rosenson R.S., Reeskamp L.F., Hovingh G.K., Kastelein J.J.P., Rubba P., Ali S., Banerjee P., Chan K.C., Gipe D.A. (2020). Evinacumab for Homozygous Familial Hypercholesterolemia. N. Engl. J. Med..

[14] Wang Y., Gusarova V., Banfi S., Gromada J., Cohen J.C., Hobbs H.H. (2015). Inactivation of ANGPTL3 reduces hepatic VLDL-triglyceride secretion. J. Lipid Res..

[15] Luo F., Das A., Fang Z. (2021). Evinacumab for Homozygous Familial Hypercholesterolemia. N. Engl. J. Med..

[16] Ando Y., Shimizugawa T., Takeshita S., Ono M., Shimamura M., Koishi R., Furukawa H. (2003). A decreased expression of angiopoietin-like 3 is protective against atherosclerosis in apoE-deficient mice. J. Lipid Res..

[17] Lee E.C., Desai U., Gololobov G., Hong S., Feng X., Yu X.C., Gay J., Wilganowski N., Gao C., Du L.L. (2009). Identification of a new functional domain in angiopoietin-like 3 (ANGPTL3) and angiopoietin-like 4 (ANGPTL4) involved in binding and inhibition of lipoprotein lipase (LPL). J. Biol. Chem..

[18] Bergmark B.A., Marston N.A., Bramson C.R., Curto M., Ramos V., Jevne A., Kuder J.F., Park J.G., Murphy S.A., Verma S. (2022). Effect of Vupanorsen on Non-High-Density Lipoprotein Cholesterol Levels in Statin-Treated Patients with Elevated Cholesterol: TRANSLATE-TIMI 70. Circulation.

[19] Stefanutti C., Chan D.C., Di Giacomo S., Morozzi C., Watts G.F. (2022). Long-Term Efficacy and Safety of Evinacumab in Patients with Homozygous Familial Hypercholesterolemia: Real-World Clinical Experience. Pharmaceuticals.

[20] Rosenson R.S., Gaudet D., Ballantyne C.M., Baum S.J., Bergeron J., Kershaw E.E., Moriarty P.M., Rubba P., Whitcomb D.C., Banerjee P. (2023). Evinacumab in severe hypertriglyceridemia with or without lipoprotein lipase pathway mutations: A phase 2 randomized trial. Nat. Med..

[21] Yue P., Tanoli T., Wilhelm O., Patterson B., Yablonskiy D., Schonfeld G. (2005). Absence of fatty liver in familial hypobetalipoproteinemia linked to chromosome 3p21. Metabolism.

[22] Inaba T., Matsuda M., Shimamura M., Takei N., Terasaka N., Ando Y., Yasumo H., Koishi R., Makishima M., Shimomura I. (2003). Angiopoietin-like protein 3 mediates hypertriglyceridemia induced by the liver X receptor. J. Biol. Chem..

[23] Ruscica M., Macchi C., Fogacci F., Ferri N., Grandi E., Rizzoli E., D’Addato S., Borghi C., Cicero A.F., Brisighella Heart Study G. (2019). Angiopoietin-like 3 and subclinical peripheral arterial disease: Evidence from the Brisighella Heart Study. Eur. J. Prev. Cardiol..

[24] Ferri N., Ruscica M. (2016). Proprotein convertase subtilisin/kexin type 9 (PCSK9) and metabolic syndrome: Insights on insulin resistance, inflammation, and atherogenic dyslipidemia. Endocrine.

[25] Schulz R., Schluter K.D., Laufs U. (2015). Molecular and cellular function of the proprotein convertase subtilisin/kexin type 9 (PCSK9). Basic. Res. Cardiol..

[26] Brown M.S., Goldstein J.L. (1997). The SREBP pathway: Regulation of cholesterol metabolism by proteolysis of a membrane-bound transcription factor. Cell.

[27] Lakoski S.G., Lagace T.A., Cohen J.C., Horton J.D., Hobbs H.H. (2009). Genetic and metabolic determinants of plasma PCSK9 levels. J. Clin. Endocrinol. Metab..

[28] Theocharidou E., Papademetriou M., Reklou A., Sachinidis A., Boutari C., Giouleme O. (2018). The Role of PCSK9 in the Pathogenesis of Non-alcoholic Fatty Liver Disease and the Effect of PCSK9 Inhibitors. Curr. Pharm. Des..

[29] Ruhanen H., Haridas P.A.N., Jauhiainen M., Olkkonen V.M. (2020). Angiopoietin-like protein 3, an emerging cardiometabolic therapy target with systemic and cell-autonomous functions. Biochim. Biophys. Acta Mol. Cell Biol. Lipids.

[30] Burks K.H., Xie Y., Gildea M., Jung I.H., Mukherjee S., Lee P., Pudupakkam U., Wagoner R., Patel V., Santana K. (2024). ANGPTL3 deficiency impairs lipoprotein production and produces adaptive changes in hepatic lipid metabolism. J. Lipid Res..

[31] Xu Y.X., Redon V., Yu H., Querbes W., Pirruccello J., Liebow A., Deik A., Trindade K., Wang X., Musunuru K. (2018). Role of angiopoietin-like 3 (ANGPTL3) in regulating plasma level of low-density lipoprotein cholesterol. Atherosclerosis.

[32] Brown M.S., Goldstein J.L. (1999). A proteolytic pathway that controls the cholesterol content of membranes, cells, and blood. Proc. Natl. Acad. Sci. USA.

[33] Wang X., Sato R., Brown M.S., Hua X., Goldstein J.L. (1994). SREBP-1, a membrane-bound transcription factor released by sterol-regulated proteolysis. Cell.

[34] Ruhanen H., Haridas P.A.N., Minicocci I., Taskinen J.H., Palmas F., di Costanzo A., D’Erasmo L., Metso J., Partanen J., Dalli J. (2020). ANGPTL3 deficiency alters the lipid profile and metabolism of cultured hepatocytes and human lipoproteins. Biochim. Et Biophys. Acta Mol. Cell Biol. Lipids.

[35] Jeong H.J., Lee H.S., Kim K.S., Kim Y.K., Yoon D., Park S.W. (2008). Sterol-dependent regulation of proprotein convertase subtilisin/kexin type 9 expression by sterol-regulatory element binding protein-2. J. Lipid Res..

[36] Sun H., Samarghandi A., Zhang N., Yao Z., Xiong M., Teng B.B. (2012). Proprotein convertase subtilisin/kexin type 9 interacts with apolipoprotein B and prevents its intracellular degradation, irrespective of the low-density lipoprotein receptor. Arterioscler. Thromb. Vasc. Biol..

[37] Bini S., D’Erasmo L., Minicocci I., Di Costanzo A., Tramontano D., Pomanti G., Covino S., Arca M., Pecce V. (2023). ApoB secretion and intracellular lipid content are modulated by ANGPTL3 and PCSK9 in HEPG2 cells. Atherosclerosis.

[38] Mehlem A., Hagberg C.E., Muhl L., Eriksson U., Falkevall A. (2013). Imaging of neutral lipids by oil red O for analyzing the metabolic status in health and disease. Nat. Protoc..

[39] Canavesi M., Baldini N., Leonardi A., Sironi G., Bellosta S., Bernini F. (2004). In vitro inhibitory effect of lercanidipine on cholesterol accumulation and matrix metalloproteinases secretion by macrophages. J. Cardiovasc. Pharmacol..

[40] Pinzon Grimaldos A., Pacella I., Bini S., Tucci G., Cammarata I., Di Costanzo A., Minicocci I., D’Erasmo L., Arca M., Piconese S. (2022). ANGPTL3 deficiency associates with the expansion of regulatory T cells with reduced lipid content. Atherosclerosis.

